# Local dispersal of palaearctic *Culicoides* biting midges estimated by mark-release-recapture

**DOI:** 10.1186/s13071-015-0658-z

**Published:** 2015-02-08

**Authors:** Georgette Kluiters, Harry Swales, Matthew Baylis

**Affiliations:** Institute of Infection and Global Health, Liverpool University Climate and Infectious Diseases of Animals (LUCINDA) Group, University of Liverpool, Leahurst Campus, Neston, Cheshire UK; National Institute for Health Research, Health Protection Research Unit in Emerging and Zoonotic Infections, University of Liverpool, Neston, UK

**Keywords:** Bluetongue, *Culicoides obsoletus*, Active dispersal, Flight, Fluorescent dust, Marking, MRR, Obsoletus group, Passive dispersal

## Abstract

**Background:**

Farm to farm movement of *Culicoides* midges is believed to play a critical role in the spread of bluetongue (BT), Schmallenberg and other midge-borne diseases. To help understand and predict the spread of diseases carried by midges, there is a need to determine their dispersal patterns, and to identify factors contributing to the direction taken and distance travelled.

**Methods:**

The dispersal of Obsoletus Group members was studied on 19 farms around Bala, north Wales. Field-collected *Culicoides* were trapped in a black-light (OVI) trap and self-marked in the collecting vessel, using micronized fluorescent dust. *Culicoides* were released at a central farm and OVI traps set on 18 surrounding farms, at distances of 1 to 4 km. The study was repeated using six colours of fluorescent dust over an 18 day period.

**Results:**

An estimated 61,062 (95% CI = 56,298-65,830) marked *Culicoides* were released during the study and 12 (0.02%) *Culicoides* were recaptured. Of the females recaptured, six were *C. obsoletus/scoticus*, two *C. dewulfi*, two *C. pulicaris* and one *C. festivipennis*. The male was *C. obsoletus*. Recaptures occurred 1–2.5 km from the release site, with greatest numbers at 2.5 km. Most recaptures were 2 nights post-release; none were more than 3 nights post-release. Two females were recovered at 1.5 km on the night of release and one male at 1 km two nights post-release. The mean distance travelled (MDT) for males was 1 km, females was 2.21 km, and all recaptured *Culicoides* was 2.15 km. Recaptures were made both downwind and upwind of the prevailing wind direction during the trapping periods, highlighting possible passive and active dispersal of *Culicoides* between farms.

**Conclusions:**

This is the first study to demonstrate farm to farm movement of the main Palaearctic BT vector species, the Obsoletus Group. Such movement has disease control implications in terms of the vectoral movement of disease between farms. The results suggest that *Culicoides* control measures applied at an infected farm (trapping or killing *Culicoides*) will reduce risk of spread to neighbouring farms by lessening the number of *Culicoides* dispersing from that farm, as well as reducing transmission at the source farm itself.

## Background

Since its emergence in northern Europe, bluetongue (BT) has spread to regions where the main Mediterranean vector species, *Culicoides imicola* Kieffer, is absent. Two vector groups, four members of the Avaritia subgenus (*C. chiopterus*, *C. dewulfi*, *C. obsoletus*, *C. scoticus*) and Pulicaris Group (*C. pulicaris* and *C. punctatus*) have been implicated as virus vectors in these regions [1]. Relatively little is known about the ecological characteristics of the newly implicated vector species [2], or indeed those believed to be non-vectors, this includes their flight behaviour [1], yet this is critical for determining the distance over which an insect may transmit a disease agent [3] and can be used to determine the size of the area over which control, such as movement restrictions or insecticidal treatment, should be applied. Knowledge of dispersal potential is an essential aspect of modelling arbovirus disease spread, therefore, there is a need to determine the dispersal patterns of northern European vector *Culicoides* species, in particular the distance over which midges fly during a set period; and to identify factors that contribute to the direction and flight distance.

Modelling of disease outbreaks suggests that long-range dispersal over land is not a common phenomenon and does not contribute to the spread of arbovirus disease [4,5], with the majority of bluetongue cases occurring within 5 km of the previous case during the 2006 European BT outbreak [6]. It is therefore of utmost importance to consider short-range dispersal, including active dispersal to find food, a mate, or an oviposition site [7].

Very few studies have investigated short distance *Culicoides* dispersal, with the most recent work undertaken in 2010 in Denmark [8], followed by work in the US in the 1980s [8-10]. Although there are a number of techniques which can be used to determine dispersal, the most commonly employed method is the mark-release-recapture (MRR) procedure. Here a large number of insects are trapped and mass-marked before being released at a central location and an attempt made to recapture those individuals at known distances from the release site.

There are a number of methods available to mark *Culicoides* for MRR studies, including radio isotopes [11], fluorescent dusts [8,9], paints [12] or dyes [13]. Dusts are the most commonly used materials for externally marking a variety of insects [14] and have the benefit of being able to mark a large number of small insects easily. The dusts used by Lillie *et al.* [7,9] and Brenner *et al*. [10] however, during their studies on *Culicoides* dispersal are no longer available, and although Kirkeby *et al*. [8] could identify fluorescein isothiocyanate using ELISA plate scanning, it is difficult to see by eye and may also be removed from *Culicoides* by the addition of ethanol to samples, so storage of these samples over time is unfeasible, unless samples are frozen.

This paper is one of a pair of companion papers, the first of which validates the use of Brilliant General Purpose fluorescent dusts and highlights the use of a self-marking method for *Culicoides* [15].

Although a number of MRR studies have been undertaken in the US, only one has been undertaken on European BT vector species, and no studies have been undertaken within a landscape consisting of a range of neighbouring farms to determine dispersal between them. A recent study in Denmark investigated a novel technique to mark *Culicoides* in the field, using fluorescein isothiocyanate, and successfully recaptured marked Pulicaris Group members, allowing them to quantify the movement of this species group between farms. Although Kirkeby *et al*. [8] highlight movement of Pulicaris Group members up to 1.75 km from their release site, members of the main European BT vector species, the Obsoletus Group, were not successfully recaptured away from the release site. There is, therefore, still a need to determine this main vector species’ dispersal patterns, particularly the distance over which these midges fly during a set period; and to identify factors that contribute to the direction and flight distance.

Here we investigate the dispersal of British *Culicoides* biting midges around a central farm in the Bala region of North Wales using MRR techniques. Specific objectives included determining the mean distance travelled over a specified period of time and determining whether *Culicoides* actively disperse between farms in the region.

## Methods

### Marking method

Kluiters *et al*. [15] investigated the use of Brilliant General Pigment (BGP, Brilliant Group, Inc., San Francisco, USA) micronized fluorescent dusts in marking *Culicoides* for dispersal studies, using a series of 11 laboratory studies in July 2010. The studies covered three areas of interest:

1. *Investigation of dust properties*: solubility in water, 10% detergent solutions and ethanol, dust adherence to *Culicoides*, dust transfer to the environment;
2. *Effects of dust on Culicoides*: dust toxicity, impact on behaviour, transfer of dust between *Culicoides*;
3. *Dust application*: application using a fine brush and gauze, injection of dust using a syringe and vacuum flask, pre-dusting trapping pots as a method of self-marking.

Full details regarding the studies undertaken on the fluorescent dusts can be seen in the companion paper [15].

### Field sampling

This study was undertaken during July 2011, in the Welsh province of Bala, situated in Snowdonia National Park. This area primarily consists of extensive sheep and beef cattle farming, with the landscape very hilly and comprised of a mixture of forests and field.

**Figure 1:**
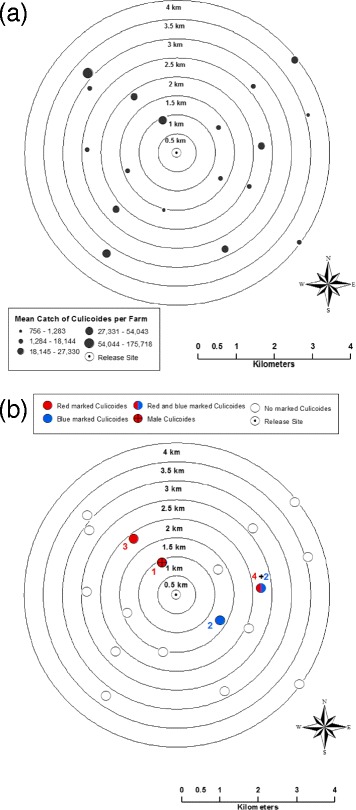
**The spatial distribution of** ***Culicoides*** **catches during a mark-release-recapture study in Bala in July 2011; where** **a)** **Spatial variation in the total trap catches of** ***Culicoides*** **(both marked and unmarked) on farms with Onderstepoort black light traps set to recapture released** ***Culicoides*** **; b)** **Spatial distribution of recaptured marked** ***Culicoides*** **, highlighting the numbers of, and colour of marking agent on, the recaptured midges.**

The study was undertaken just north of Bala Lake, where previous studies had collected large numbers of *Culicoides* on 34 farms within a 6x6 km area [16,17]. ArcGIS Desktop 10 (ESRI, Redlands, California, USA) was used to create concentric buffer zones of 0.5 km around the central-most farm within the Bala field-site, to a radius of 4 km from the central farm (Figure 1). Eighteen of the 24 other study farms in the area, which fell within the 4 km buffer zones, were randomly selected to participate within the study. All farms were recruited via personal contact.

#### Mark-release-recapture

Six preliminary catches were undertaken on the 3 farms that were to be used to collect unmarked *Culicoides* (collection farms). Catches were cleaned and counted in order to estimate the number of *Culicoides* collected from each farm, and therefore released during each replicate of the experiment.

*Culicoides* specimens for marking were live-trapped on 3 farms (including the release site) using Onderstepoort-down draught type black light (OVI) traps containing a 23 cm 8 W black light bulb. Normal collecting beakers were replaced with gauze-bottomed beakers pre-dusted with 1 g of Brilliant General Pigment micronized fluorescent dust, following the methods of Kluiters *et al*. [15]. Each trap was operated overnight and the self-marked *Culicoides* were released at the release site (see Figure 1) at 0900–1000 hrs the following morning.

OVI traps were positioned on all farms except the release site, and were located near feeding (host) and breeding sites, while avoiding other light sources to limit interference. *Culicoides* were trapped into 200 ml water and a small amount of washing up liquid, to break the surface tension. As marked *Culicoides* were released in the morning, traps were run 24 hours a day.

Every 24 hrs for 3 days following release, collecting vessels were changed in order for the time period when marked *Culicoides* were trapped to be determined. This MRR cycle was repeated five more times, so as to maximise the chance of recapturing the released *Culicoides.* Six colours (Pink, Green, Red, Blue, Orange, and Yellow) of micronized fluorescent dust were used chronologically to allow for replications of the MRR experiment, which therefore ran for a total of 18 days between 5^th^ to 23^rd^ July. The temperature, humidity, precipitation, wind speed and direction for this period can be seen in Table 1. This data was collected at the release site using APRS World, LLC (Winona, USA) data logging equipment. Wind speed, direction and humidity were averaged over each 24 hr period (beginning at 9 am).

**Table 1:** **Weather variables for the Bala region of north Wales from 5** ^**th**^ **to 23** ^**rd**^ **July 2011**

**Date**	**Temperature (°C)**			**Precipitation (mm)**	**Humidity (%)**	**Wind**	
	**Maximum**	**Minimum**	**Average**			**Speed (m/s)**	**Direction**
05/07/2011^a^	20	14	17	0.8	65	4.72	SE
06/07/2011^a^	20	13	16	0.6	67	3.61	SW
07/07/2011^a^	20	11	16	2	71	5	SW
08/07/2011^ab^	18	8	13	3	78	4.44	S
09/07/2011^b^	20	12	16	0	65	3.61	NW
10/07/2011^b^	17	12	14	15	78	2.22	NW
11/07/2011^bc^	19	8	14	0	69	2.22	NW
12/07/2011^c^	20	11	16	0	65	2.22	E
13/07/2011^c^	18	15	12	0	57	2.78	N
14/07/2011^cd^	21	7	14	0	61	3.33	NW
15/07/2011^d^	22	9	16	0.4	64	2.78	S
16/07/2011^d^	19	13	16	2	72	4.72	SW
17/07/2011^de^	15	11	13	6	87	3.89	W
18/07/2011^e^	17	13	15	0.4	78	5	W
19/07/2011^e^	16	13	14	0.2	77	4.17	NW
20/07/2011^ef^	17	14	11	0	66	3.06	NW
21/07/2011^f^	18	12	15	0	75	2.78	N
22/07/2011^f^	18	11	14	1	73	1.94	N
23/07/2011^f^	17	7	12	0	67	3.61	NW

Differing colours of micronized fluorescent dusts were used consecutively during the study, whereby ^a^indicates pink, ^b^is green, ^c^is red, ^d^is blue, ^e^is orange, and ^f^is yellow. The first date for each dust replicate indicates the day that marked individuals were released, with the 3 subsequent days representing recapture days.

#### Sorting and storing

*Culicoides* from the daily caches were examined for the presence of fluorescent dusts in the field, before being stored in 70% ethanol and later examined under a stereomicroscope. Marked individuals were counted and the recapture location and date recorded, before being further identified to species level. For female members of the Obsoletus Group, *C. obsoletus* and *C. scoticus* were not separated from one another. The number of *Culicoides* trapped during the study at each location, as well as the numbers trapped during preliminary trapping, was determined by sub-sampling the catches, but, in the interests of time, the species composition was not recorded.

#### Data analyses

The number and location of recaptured specimens was used to determine the mean distance travelled (MDT) by males and females, using the methods of Lillie *et al*. [9]. The number of recaptured *Culicoides* was corrected to account for unequal trapping areas and unequal trap density in each of the concentric distance bands. The proportion of the total trapping area occupied by each concentric ring was calculated, and multiplied by the total number of traps (T_t_) used in order to determine the number of traps needed in each trapping area for equal trap density (Table 2). The number of midges recaptured at a given distance from the release site was then corrected by multiplying by the ratio of *Expected number of traps/Actual number of traps*.

**Table 2:** **Determining the expected numbers of traps in each concentric ring of a mark-release-recapture experiment**

**Radius of concentric ring (km)**	**Area of circle (km** ^**2**^ **)** ^***Aft***^	**Area of concentric ring (km** ^**2**^ **)** ^***As***^	**Actual number of traps**	**Expected number of traps**
**0 - 0.5**	0.79	0.79	0	0.28
**0.5 - 1.0**	3.14	2.36	1	0.84
**1.0 - 1.5**	7.07	3.93	3	1.41
**1.5 - 2.0**	12.57	5.50	2	1.97
**2.0 - 2.5**	19.63	7.07	4	2.53
**2.5 - 3.0**	28.27	8.64	3	3.09
**3.0 - 3.5**	38.48	10.21	2	3.66
**3.5 - 4.0**	50.27	11.78	3	4.22
**Total**	**160.22**	**50.27** ^***At***^	**18** ^***Tt***^	**18**

*Area of circle* is the area of the circle contained within the outer limit of the concentric ring. *Area of concentric ring (A*_*s*_ *)* is the area within the inner and outer limits of the ring. *Actual number of traps* is the number of traps within each concentric ring. *Expected number of traps*, A_s_ / A_t_ × T_t_, is the number of traps required in each concentric ring to achieve equal density in all rings; where *T*_*t*_ is the total number of traps and *A*_*t*_ is the total trapping area.

The corrected data were used to estimate the mean distance travelled by marked specimens during the release night, one night post release and two nights post release. Additionally, the corrected data were pooled for the duration of the experiment to determine the MDT during this period.

$$ MDT = \frac{{\displaystyle \sum}\left( Expected\  no.\kern0.5em  recovered \times Distance\right)}{{\displaystyle \sum } Expected\  no.\kern0.5em  recovered} $$

## Results

### Marking method

Marked midges remained distinguishable for their entire life; dusts did not transfer from marked to unmarked individuals or the environment; the mortality rate of marked midges did not differ from controls under laboratory conditions; and, importantly for trapping and storing *Culicoides*, the dust did not dissolve or wash off in either ethanol or water. The dusts were shown to be a fast and reliable method for marking *Culicoides* in the field and did not appear to influence flight behaviour in the laboratory. A self-marking method for MRR studies of *Culicoides* was therefore devised using the fluorescent dusts, by coating the inside of gauze-bottomed trapping containers with the dust prior to trapping them. For further details see Kluiters *et al*. [15].

### Field sampling

An estimated 10,177 (95% CI = 9,383-10,972) marked *Culicoides* were released per day (61,062 [95% CI = 56,298-65,830] during the total study). By sub-sampling the catches, an estimated total of 501,094 *Culicoides* were trapped in the recapture traps (Table 3), while the maximum catch per night varied on farms between 72 and 33,693 *Culicoides*. The spatial variation in overall catches between the farms can be seen in Figure 1a.

**Table 3:** ***Culicoides*** **trapped in the recapture traps during a mark-release-recapture experiment in the Bala region of north Wales**

**Farm ID**	**Maximum** ***Culicoides***	**Mean** ***Culicoides***	**Total** ***Culicoides***	**Recaptured** ***Culicoides***
**A1**	33694	8861	175718	0
**A6**	2550	1162	19966	0
**B1**	2458	974	13544	0
**B2**	7494	1438	27330	3 R
**B5**	3100	538	9650	0
**C1**	1647	1317	17117	0
**C3**	7310	2143	40721	1 R
**C4**	5781	1848	16762	0
**C5**	4032	1650	23098	4 R + 2 B
**C6**	142	70	1283	0
**D2**	2835	1735	18144	0
**D4**	6016	1145	14881	2 B
**D5**	1161	367	8824	0
**E1**	2353	1435	24613	0
**E3**	72	40	756	0
**F1**	9979	3002	54043	0
**F4**	2802	913	19527	0
**F6**	2142	857	15117	0
**Total**	**95568**	**29496**	**501094**	**12**

R indicates *Culicoides* marked with red fluorescent dust; B indicates *Culicoides* marked with blue fluorescent dust.

**Figure 2:**
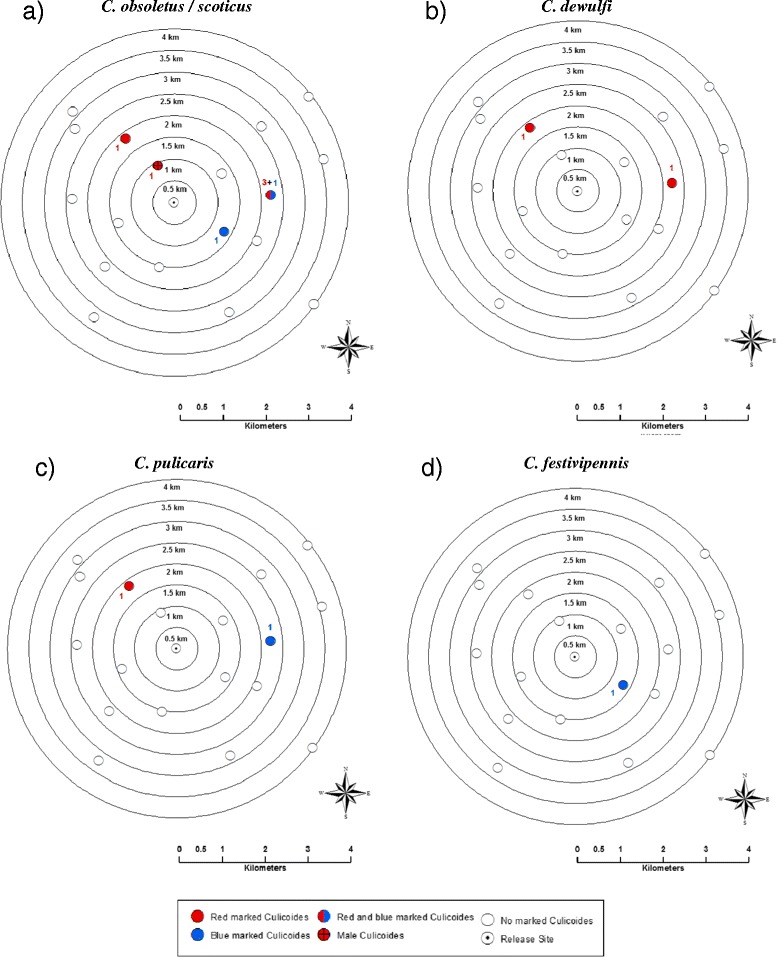
**Spatial distribution of the species of** ***Culicoides*** **recaptured during the mark-release-recapture in Bala; where** **a)** ***C obsoletus/scoticus*** **;** **b)** ***C. dewulfi*** **;** **c)** ***C. pulicaris*** **;** **d)** ***C. festivipennis*** **.** Numbers indicate the number of the species caught at each site.

A total of 12 (0.02%) marked *Culicoides* were recaptured, 8 were marked with red fluorescent dust and 4 were marked with blue (Table 3). Figure 1b shows the spatial distribution of the recaptured *Culicoides* by colour of dust. No recaptures were made of *Culicoides* marked with other colours of dust. Of the females, six were *C. obsoletus/scoticus*, 2 *C. dewulfi*, 2 *C. pulicaris* and 1 *C. festivipennis*. All females collected were nulliparous. The male was *C. obsoletus* s.s. Figure 2 shows the spatial distribution of the recaptured midges, of each colour, by species.

The marked *Culicoides* were released on a farm with 4,000 sheep and 350 beef cattle present, at an altitude of 223 m. Recaptures were made on two farms with sheep and beef cattle present (550 and 30; and 500 and 20 respectively), one farm with only sheep present (144), and one site with no animals, at altitudes of 287 m, 214 m, 235 m and 172 m respectively.

The majority of recaptures were 2–2.5 km from the release site and the most numerous recaptures were 2 nights post-release. No marked *Culicoides* were trapped more than 3 nights post-release, following each dusting replicate. Eleven females were recovered up to 2.5 km from the release site, with two of these females recovered at a distance of 1.5 km on the night of release. One marked male was recovered and was trapped two nights post-release at a distance of 1 km. No *Culicoides* were recaptured at distances greater than 2.5 km (Table 4).

**Table 4:** **Mean distance travelled (km) from release site by individual** ***Culicoides*** **species during a mark-release-recapture experiment in the Bala region of north Wales**

	**Nights post-release**			
**Species**	**1**	**2**	**3**	**Entire experiment**
*C. obsoletus/scoticus*	1.5	2.2	2.5	2.17
*C. dewulfi*	-	2	2.5	2.2
*C. pulicaris*	-	2.2	-	2.2
*C. festivipennis*	1.5	-	-	1.5
*C. obsoletus* Male	-	1	-	1
**All** ***Culicoides***				
Female	1.5	2.15	2.5	2.21
Male & Female	1.5	1.79	2.5	2.15

**Figure 3:**
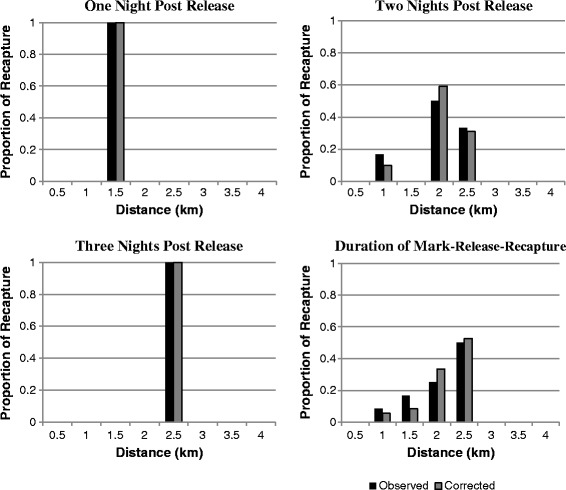
**Histograms of observed and corrected data at three post-release times and pooled for the entire mark-release-recapture period.**

The *Culicoides* dispersed to greater distances as the post-release time increased (Figure 3). Based on the corrected data, the *Culicoides* travelled a mean distance of 1.5 km during the release night (Table 4). The MDT increased to 1.79 km for two nights post release (2.15 km for females only) and 2.5 km for three nights post-release. There were no *Culicoides* recaptured after 3 nights post- release. A change in the rate of dispersal was noted as the time post release increased. The MDT for the first 24 hrs after release was 1.5 km. In the second 24 hrs, the MDT increased by 0.65 km and in the third 24 hours by 0.33 km.

The MDT for all *Culicoides* through the entire mark-release-recapture was 2.15 km. The MDT for the male was 1 km, whereas for females it was 2.21 km. Table 4 shows the MDT by each species individually during the MRR experiment.

## Discussion

This study is the first to successfully demonstrate the dispersal of the Obsoletus Group members between farms. Kirkeby *et al*. [8] marked and released 607 specimens of the Obsoletus Group on a cattle farm in Denmark yet were unsuccessful in recapturing members of this Group in any of the surrounding traps (other than the release site), highlighting a continued need for the dispersal of the main Palaearctic BT vector species to be investigated.

The experiments undertaken to validate the use of the fluorescent dusts prior to the field trial highlight that these dusts are a suitable marking agent for *Culicoides* in either a laboratory or field setting. The dusts were shown to be a fast and reliable method for marking *Culicoides* as part of a self-marking method, by baiting the inside of gauze-bottomed trapping containers with the dust prior to trapping them. The use of such a method is likely to reduce damage and death of individuals that can occur by applying the dust after capture.

With such low numbers of marked *Culicoides* identified however, it would have been useful to test the sensitivity of identifying marked *Culicoides* from the catches prior to the field study. This could have been achieved by adding a known number of marked insects to catches of unmarked *Culicoides* stored in ethanol, and determining how many of these marked individuals were identified by the researchers involved when sorting through those insects.

In other studies, less than 1% of the number of insects released in a MRR study is typically recovered [18]. Kirkeby *et al*. [8] recaptured 0.75% of *Culicoides* released at sites away from the release point, all of which were Pulicaris Group species. Lillie *et al*. [9] recaptured 0.5% of *C. variipennis* in Colorado, and 1.5% of *C. mississippiensis* when undertaking a MRR study in Florida [7]. An exception to this was seen in the work of Brenner *et al*. [10] where 13% of *C. mohave* were recovered in southern California, due to the lack of vegetation in the desert environment where the study took place. The landscape in Bala comprises a mixture of forest and field and is an undulating landscape with many natural barriers and sources of water, so low observed recapture rate was expected.

No other dispersal studies on *Culicoides* have been undertaken in such an undulating or vegetation-filled environment. Previous studies focus on dispersal in saltmarsh regions of Florida [7], the desert of Southern California [10], the South Platte River drainage system in Colorado [9], and an open field-landscape in Denmark [8]. However we successfully highlight the dispersal of *Culicoides* between neighbouring farms in this environment.

Our data support the dispersal, or gradual movement, of *Culicoides* away from a release site. The MDT of the *Culicoides* during the first release night is comparable to that seen for *C. mississippiensis* [7], where most individuals were taken at up to 1.5 km at 24 hrs post release. *Culicoides* were not recaptured as far as the 4 km observed for *C. variipennis* over a 36 hr period in Colorado [9]. Our estimates of flight range are based on a small number of *Culicoides* recaptured and it is therefore not possible to determine if differences exist in flight distances between species. Although *C. festivipennis* was not found further than 1.5 km from the release site, this species is far less abundant than members of the Obsoletus Group, so we would expect to recapture very comparatively fewer [16]. We are able to show that recaptured Obsoletus Group females, as well as *C. pulicaris,* are able to disperse a minimum of 2.5 km in 48 hrs.

Data obtained from the MRR study show that *Culicoides* travel between farms in this region. Such movement has disease control implications in terms of the vectoral movement of disease between farms. Hocking [3] stated that the flight range of an insect can be used to determine the distance over which that insect may transmit a disease agent, and recommended treating an area equivalent to the square of the MDT for effective control following a single application. The MDT of the *Culicoides*, and the fact that they appear to freely move from farm to farm, even in such a heterogeneous landscape, highlights the unfeasibility of this method of control.

Previous studies have noted an abrupt decline in recapture following one night post-release [19,20]. The change in the rate of dispersal as the time post-release increased is likely the result of multi-directional flight patterns, physiological changes, or environmental influences. Females would have been less likely to have been trapped as time elapsed, if the need for a blood meal had been fulfilled early on. Instead, they would be searching for an oviposition site rather than a host from which to take a blood meal. This decline in dispersal, or recapture, rates with time has previously been attributed to midges dispersing into areas of low trap density [9], as well as mortality, behavioural changes, or a combination of these factors [21].

The directions that the *Culicoides* took may be related to the topography of the landscape (rivers, valleys etc.), but we did not explore this due to low recapture rates. *Culicoides* did not appear to disperse towards farms with larger numbers of livestock present and indeed were trapped on one premise containing no livestock. The red-marked *Culicoides* recaptured on farms to the northwest of the release farm may have been aided by wind dispersal, with the wind during the day of release heading north-westerly. Similarly the red-marked individuals trapped on a farm easterly from the release site at 2 days post-release may have been influenced by the easterly wind recorded a day following their release. The same cannot be said for the blue dust-marked individuals found in the east and south-east, with the wind heading northwest on the day of release before changing to the southwest, highlighting that these individuals flew upwind. These findings may be explained by Sedda *et al*. [6] who considered that during the European BTV-8 outbreak, upwind midge flight may be a response to wind acting as a carrier of host semio-chemicals, while downwind movement of midges was due to wind transporting the midges themselves. It is important to note, however, that our wind direction data are 24 hr averages and do not provide the temporal resolution to examine wind directions at night only, when *Culicoides* flight is most likely. Large changes in wind direction may also be caused by complex topography, such as that found in the study site, therefore the wind direction and speed at the release site may vary significantly from that in other areas of the field site.

The recapture of a male *C. obsoletus* at a distance of 1 km from the release site was unexpected. Male recapture is rare in MRR studies with no *C. mohave* recaptured in southern California, despite the homogenous landscape [10], and only 2 male *C. mississippiensis* recaptured 0.5 km from their release point by Lillie *et al*. [7]. The maximum distance travelled by male *Culicoides*, prior to this study, was by *C. variipennis* which travelled 0.8 km in Colorado [9]. The number of males trapped during entomological trapping regimes at light is generally less than 10% of the females trapped. When considering males do not take a blood meal, may remain closer to breeding sites than females [22], and do not appear to be attracted to light traps [23], it would be expected that fewer males would be recaptured in a MRR study.

## Conclusions

This study is the first to demonstrate the active dispersal of the *Culicoides* Obsoletus Group from farm to farm. Although the recapture rate was small, we have provided evidence that species of the Obsoletus Group are able to disperse 2.5 km or more, with males able to disperse to a distance of 1 km in 24 hrs. The results suggest that *Culicoides* control measures applied at an infected farm (which trap or kill *Culicoides*) will reduce risk of spread to neighbouring farms by lessening the number of *Culicoides* dispersing from an infected farm, as well as reducing transmission at the source farm itself.

## Abbreviations

BT: Bluetongue
MDT: Mean distance travelled
MRR: Mark-release-recapture
OVI: Onderstepoort veterinary institute (down-draught black light traps)
